# Public sanitation interventions and household clean energy adoption: Evidence from China’s renovating water supply and toilets

**DOI:** 10.1371/journal.pone.0333630

**Published:** 2025-10-06

**Authors:** Ping Wang, Lijiao Liu

**Affiliations:** School of Economics, Southwestern University of Finance and Economics, Chengdu, China; Gadjah Mada University Faculty of Medicine, Public Health, and Nursing: Universitas Gadjah Mada Fakultas Kedokteran Kesehatan Masyarakat dan Keperawatan, INDONESIA

## Abstract

By 2021, approximately 2.3 billion people worldwide still relied on polluting fuels and technologies for cooking. Such harmful fuels pose serious risks to both health and the environment, contributing to health issues and constraining economic activities. Based on the China Health and Retirement Longitudinal Study data, this study investigates whether and how the renovation of tap water and sanitary toilets affects clean energy adoption among rural households in China by utilizing a Probit model. The results demonstrates that the probability of rural households using clean energy for cooking significantly increased by 8.0 percentage points after undergoing the renovation of tap water and sanitary toilets. These effects are particularly pronounced in households with male heads and low-income households. Mechanism analysis suggests that the renovation of tap water and sanitary toilets significantly elevates educational levels, strengthens cognitive abilities, and fosters the accumulation of human capital, while also raising environmental awareness, thereby driving the utilization of clean energy sources. These findings underscore the significance of sanitation improvements in driving energy transition in rural areas of China. For policymakers focusing on sanitation facilities and socioeconomic development in developing countries, such as China, investments in sanitation infrastructure can yield broad benefits beyond public health, fostering human capital accumulation, environmental awareness, and energy transition. These outcomes have significant implications for long-term sustainable development goals.

## 1. Introduction

Globally, the adoption of clean energy adoption has been accelerating; yet, residential usage remains relatively low. In 2021, approximately 2.3 billion people worldwide still relied on polluting fuels for cooking. China, one of the world’s largest emitters of energy and a significant energy consumer, recorded a cooking clean energy utilization rate of only 79% [1]. Due to the effects of COVID-19 and the sharp rise in energy prices, the International Energy Agency estimates that about 100 million people who had recently transitioned to clean cooking may revert to traditional biomass practices [2]. Encouragingly, countries such as China, Kenya, and India have made notable advances through strengthened political commitment, effective public communication, and sustained financial investment. For instance, China’s coal-to-electricity conversion plan not only implements subsidies for new technologies but also prohibits household coal usage [3]. This comprehensive strategy effectively encourages residents’ adoption of clean energy. However, given the current pace of progress in energy access or the policies of various countries, achieving the universal goal of clean cooking by 2030 remains a considerable challenge. How to accelerate the development of clean and low-carbon energy while phasing out traditional biomass energy has thus become a pressing global priority.

The existing literature has shown that the widespread use of traditional energy sources such as coal, straw, and firewood in households has had a serious impact on human life and health [4]. Numerous studies have confirmed the negative consequences of non-clean energy use, such as climate change, air pollution, low birth weight, acute respiratory infections, gender inequality, and decreased subjective well-being [5–10]. Thus, how to drive the transition of energy use in the household sector to clean energy is the focus of current scholarly attention. A growing number of scholars have found that income, educational level, and environmental awareness are important driving factors for promoting the transition in energy use in the household sector to clean energy [11–13]. As income increases, individuals’ basic living needs are met, their demand for high living environment quality continues to increase, their demand for a green, low-carbon, and healthy life becomes stronger, and they are more motivated to use clean energy [14]. Improving education and health status can help enhance residents’ sense of environmental responsibility and environmental awareness, change pro-environmental behavior, thereby facilitating energy transformation [15,16].

Recent literature suggests that improved public sanitation services have greatly facilitated the lives of residents, and public health construction has had a positive impact on improving individual education, economic benefits, health, and reducing infectious diseases [17–20]. Some studies emphasize that improving sanitation conditions, such as the reduction of open defecation, can help alleviate diseases such as anemia, increase the average height of children, decrease healthcare expenses and produce better health outcomes [21–23]. Especially for students, public sanitation in schools can help increase their enrollment, social participation, attention, attendance rates and educational attainment [24–26]. Furthermore, improving public sanitation through government intervention can enhance individuals’ quality of life by improving their health, disgust, shame, safety, privacy, and other factors [18]. However, there is little literature on the impact of public sanitation services on household energy consumption and clean energy transition. In this context, it is worth analyzing the role of public sanitation service policies, such as the renovation of tap water and sanitary toilets, in encouraging residents to voluntarily adopt clean energy.

Based on representative data from the China Health and Retirement Longitudinal Study (CHARLS), this study examines the relationship between the construction of public sanitation services—specifically the renovation of tap water and sanitary toilets in rural China—and residents’ energy transition. The possible contributions of this study are as follows. First, by linking the renovation of tap water and sanitary toilets in rural China with household clean energy use, this study establishes a unified analytical framework to explore their association, along with potential mechanisms and heterogeneity across households. To the best of our knowledge, there is limited literature on the impact of public sanitation construction on micro-level household energy consumption or energy transition. Second, this study employs a Probit regression model to estimate the relationship between public sanitation services and household clean energy adoption, providing empirical evidence from the perspective of rural China. Third, this study reveals that the benefits of improving public sanitation services not only manifest in the improvement of the rural living environment but also promote long-term energy transition and clean energy use in households. These findings provide research support for further advancing public sanitation construction and energy transition, and offers a reference for the formulation of relevant policies.

Building on the broader context of rural public sanitation improvement and its potential socio-economic implications, this study anticipates that the renovation of rural tap water and sanitary toilets will promote households’ adoption of clean energy and contribute to energy transition. We also expect that earlier exposure to such renovation will yield stronger effects, and that these impacts may operate primarily through enhancing human capital—such as education and cognitive ability—and fostering environmental awareness. The theoretical basis and detailed development of these hypotheses are elaborated in the “Research background and theoretical hypothesis” section.

The remainder of this study is as follows: Section 2 introduces the policy background and proposes theoretical assumptions. Section 3 presents the data sources and empirical identification strategy. Section 4 shows the empirical results. Finally, the conclusion and related policy implications of this study are presented in Section 5.

## 2. Research background and theoretical hypothesis

Efficient public sanitation facilities are essential to improving the living environment, enhancing health and promoting overall wellbeing of the local residents. According to the first global guidelines on sanitation and health launched by the World Health Organization (WHO), approximately 4.2 billion people globally lack access to safely managed sanitation services, resulting in approximately 432,000 deaths each year [27]. Every $1 invested in sanitation services is repaid five-fold in lower healthcare costs, improved productivity, education, and jobs [28]. To improve sanitation services, governments and social organizations in various countries have invested substantial resources, implementing measures such as the popularization of tap water, the construction of urban sewers, the construction of sanitary toilets, and health campaigns against epidemics.

Before the reform and opening up in China, people and animals in many rural areas lived together in the same yard, where animal manure was piled up. It was very common that people had no toilets and livestock had no pens. After the reform and opening up, the use of chemical fertilizers in rural areas has increased substantially, resulting in a decrease in the demand for manure as an organic fertilizer. The problem of environmental pollution and drinking water safety caused by manure has become increasingly prominent. In 1980, the 35th United Nations General Assembly proposed launching a ten-year “International Drinking Water Supply and Sanitation” campaign (1981–1990) to address the lack of safe drinking water and sanitation facilities affecting more than half of the world’s population. The Chinese government supported this initiative, deciding to make the renovation of rural water supply and toilets an important component of national health reform and development. In 1986 and 1996, the Chinese government included targets for improving rural drinking water and constructing sanitary toilets in the “7th Five-Year Plan” and “9th Five-Year Plan” respectively. In the new century, the Chinese government continued to incorporate the upgrading of rural tap water and sanitary toilets into government planning. In the specific implementation process, the central and local governments subsidized rural households and stipulated guidelines for the implementation of rural tap water and toilet renovation projects. Subsequently, the sanitary conditions of drinking water and sanitary toilets in rural areas have greatly improved. According to statistics from the Chinese government, by the end of 2022, the penetration rate of tap water in rural areas had reached 87%, and the penetration rate of sanitary toilets had exceeded 73%.

This raises the question of how does the renovation of rural tap water and sanitary toilets affect the use of clean energy in households. In fact, the renovation of rural tap water and sanitary toilets can improve public health condition in communities, and promote the use of clean energy in households through two possible channels: human capital accumulation and environmental awareness.

Some evidence suggests that households with access to higher quantities of tap water are expected to shorten distances to water sources, and have more water available for productive activities and increase food security and nutrition [29,30]. The accessibility of tap water and sanitary toilets is linked to the decrease in the prevalence of infectious diseases and other health issues caused by poor hygiene conditions, thus comprehensively elevating the overall health residents’ status [31–33]. In addition, modernized facilities can also save residents time that would otherwise be spent fetching water from distant sources or addressing inadequate sanitation facilities [34]. The improvement in health levels and time savings can be better allocated to productive activities, including learning and skill development [35], thereby promoting the enhancement of education and cognitive abilities [26,36]. Having higher levels of education and cognitive abilities indeed implies a greater level of human capital. Individuals with higher human capital are more likely to understand the importance of clean energy and how to effectively utilize it [37,38]. They may be more receptive to environmental and sustainable energy concepts, thereby promoting the use of clean energy within their households. Therefore, the renovation of tap water and sanitary toilets can promote household adoption of clean energy by enhancing human capital.

Personally experiencing improvements in water resources and sanitation conditions often leads to a heightened sense of environmental awareness and recognition of the importance of environmental conservation. Moreover, population groups with higher human capital generally exhibit superior learning and information acquisition capabilities. This equips them to gain a more profound understanding of their living environment and recognize the vital roles that a clean environment and clean energy play in their lives. The educational and informational advantages can yield positive impacts, fostering the development of environmental awareness and promoting the adoption of sustainable behaviors [39,40]. Therefore, the renovation of tap water and sanitation facilities can not only create a clean and tidy environment but also enhance individuals’ environmental awareness by boosting human capital. The interaction between improving tap water and sanitation facilities and the enhancement of environmental awareness has resulted in positive effects, contributing to the promotion of clean energy use in households.

Based on the above discussion, this paper proposes three hypotheses to be tested. **Hypothesis 1:** The renovation of rural tap water and sanitary toilets can promote the use of clean energy in households and foster energy transformation. **Hypothesis 2:** Considering the cumulative effect, the earlier households experience the renovation of rural tap water and sanitary toilets, the higher the probability of using clean energy and the more significant the effect of promoting energy transformation. **Hypothesis 3:** The renovation of rural tap water and sanitary toilets mainly promotes the use and transformation to clean energy in households by enhancing human capital (including raising educational levels and cognitive abilities) and fostering environmental awareness.

## 3. Data and methodology

### 3.1. Data

The microdata used in this study is from the CHARLS, a nationally representative survey of individuals aged 45 and above in China [41]. The baseline wave in 2011 covered around 17,000 individuals from 10,000 households. This dataset provides rich information on household economic and demographic characteristics, as well as primary cooking fuel, thereby enabling this study to examine the patterns and transitions in household clean energy use.

**Fig 1:**
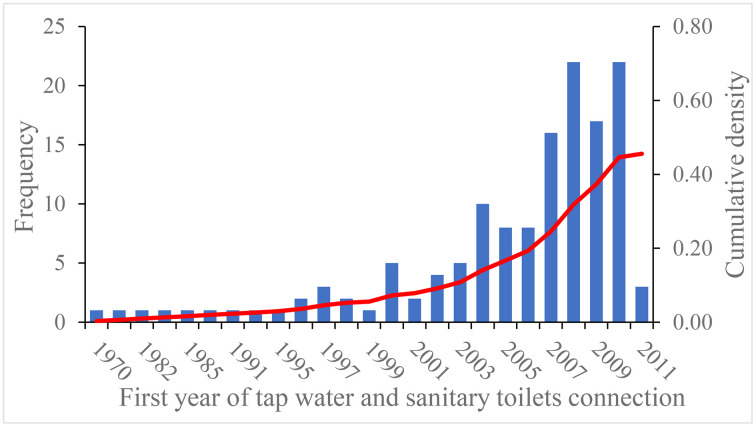
Number of new villages that connect the renovation of tap water and sanitary toilets by year.

CHARLS currently includes four waves: 2011, 2013, 2015, and 2018. In the community questionnaire of the baseline national wave in 2011, respondents were asked, “Has your village restructured the rural tap water and sanitary toilets?”. If so, they were then asked, “When was the restructuring of tap water and sanitary toilets done?”. Fig 1 shows the number of new villages renovated by tap water and sanitary toilets each year, as well as the cumulative distribution of the village-level tap water and sanitary toilets roll-out from 1970 to 2010 among 305 villages in CHARLS 2011. In 1970, less than 1% of the villages have undergone the renovation of tap water and sanitary toilets. By 2011, approximately 45.45% of villages have been restructured. According to the CHARLS, the restructuring of tap water and sanitary toilets in rural areas is relatively concentrated after 1995, which corresponds to the time dimension when China included rural environmental sanitation in the 9th Five-Year Plan in 1996.

Considering the feasibility of this study, we processed the data as follows. We extracted information from all households in the sample of 305 valid villages in four rounds of surveys from 2011 to 2018. If the household has undergone multiple rounds of surveys, only the information from the last survey round is kept. To avoid the influence of extreme values, we excluded these households whose income is equal to 0 and whose energy consumption information is missing. The income of households is deflated by the 2011 price index. Meanwhile, households with a head age less than 45 years are excluded, because CHARLS aims to collect a nationally representative sample of Chinese residents ages 45 and older, the sample under 45 years of age may be outlier or special value. In addition, to ensure the accuracy of the sample affected by the renovation of tap water and sanitary toilets, we retained residents who have been living in the village and have not undergone any relocation. We finally use multi-year mixed cross-sectional data with a total of 9684 households.

This study used publicly available secondary data from the CHARLS, conducted by the National School of Development and the Institute of Social Science Survey at Peking University. Ethical approval for the original data collection was obtained from the Institutional Review Board of Peking University (IRB00001052–11015). All participants provided informed consent prior to participation in the survey. As this research is based entirely on anonymized, publicly available secondary data, no additional ethical approval was required.

Our main outcome was household clean energy adoption obtained from CHARLS. In the questionnaire of CHARLS, the respondent was asked “What is the main source of cooking fuel?”. The main options are divided into seven categories, including coal, firewood, electricity, natural gas, marsh gas, liquefied petroleum gas, and others. If a household mainly uses energy such as natural gas, marsh gas, liquefied petroleum gas, or electricity for cooking, we define the main energy source used by the household as clean energy, and this variable was code as 1. Otherwise, if coal, straw and other energy sources are used in cooking, then the variable is equal to 0. The statistical description of variables used in this study is shown in Table 1.

**Table 1:** Statistical description of variables.

Variables	Descriptions	Mean/percentage	Std. Dev.
**Explained variable**			
Coal	Coal for cooking = 1, others cooking = 0	0.053	0.224
Firewood	Crop residue or firewood burning for cooking = 1, others cooking = 0	0.339	0.473
Gas	Gas (such as natural gas and liquefied petroleum gas) for cooking = 1, others for cooking = 0	0.352	0.478
Cleanenergy	Clean energy (such as natural gas, liquefied petroleum gas, marsh gas and electric) for cooking = 1, others for cooking = 0	0.593	0.491
**Individual characteristics of the household head**			
Age	Age (in years)	63.696	10.970
Gender	Male = 1, female = 0	0.481	0.500
Marital status	Current marital status, Married or Cohabitation = 1, otherwise = 0	0.713	0.453
Education	Years of education	4.627	3.950
Medical insurance	Having medical insurance = 1, otherwise = 0	0.959	0.199
Pension insurance	Having pension insurance = 1, otherwise = 0	0.844	0.363
**Household characteristics**			
Family size	Number of persons in household	2.820	1.622
CDR	Proportion of children aged 16 and below in family size	0.061	0.140
ODR	Proportion of the elderly aged 65 and above in family size	0.354	0.417
Lnincome	Total household income (log of RMB value)	10.059	1.394
Lnassets	Total household assets (log of RMB value)	10.843	2.185
Obs.	9680		

Based on the historical data on “renovating water supply and toilets” in rural areas, the samples were categorized into two groups: villages that have undergone the renovation of water supply and sanitary toilets and villages that have not yet implemented the renovation. On average, the data show that the samples from villages that have implemented the renovation demonstrate a higher level of clean energy adoption compared to samples from villages that have not undergone the renovation (Table 2). Additionally, these villages display reduced reliance on coal, firewood, and other traditional fuels. These differences collectively indicate that “renovating water supply and toilets” contributes to the adoption of clean energy in households and promotes the transition to clean energy in rural households. However, a simple descriptive analysis may not capture the causal effects of this reform on household clean energy usage and may only reveal a correlation. Therefore, further empirical testing is required to establish more robust causal effects.

**Table 2:** Differences in clean energy adoption among different groups.

Variables	Treat group		Control group		(Treat group- Control group)
	Mean	Std. dev.	Mean	Std. dev.	Mean difference
Coal	0.026	0.160	0.075	0.264	−0.049***
Firewood	0.302	0.459	0.370	0.483	−0.068***
Gas	0.420	0.494	0.295	0.456	0.124***
Cleanenergy	0.658	0.474	0.538	0.499	0.120***
Obs.	4433		5251		

Note: Households located in villages that have undergone the renovation of tap water and sanitary toilets are assigned to the treat group, while those in villages without such renovations form the control group. Significance levels: *p < 0.10. **p < 0.05. ***p < 0.01. Mean differences between the treatment and control groups were tested using two-sample t-tests (Stata command: ttable3).

**Fig 2:**
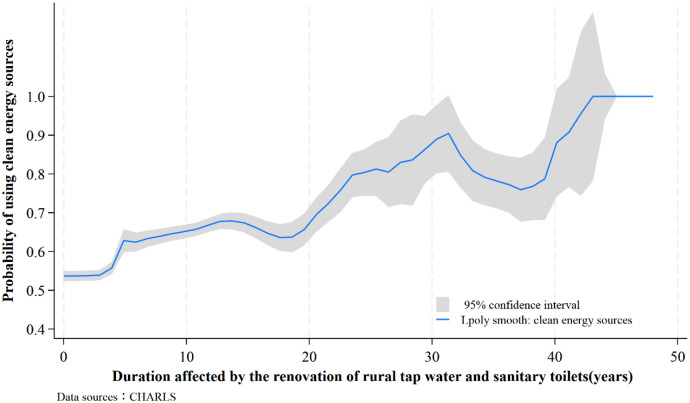
The relationship between the duration of tap water and sanitary toilet renovation and the adoption of clean energy in households.

Furthermore, it is crucial to understand that the effects of the renovating water supply and toilets on household clean energy adoption are not limited to short-term or temporary changes. Instead, they are expected to have a long-lasting impact. Fig 2 illustrates the relationship between the duration of exposure to the improving water supply and toilets and the adoption of clean energy in households. It reveals that as the duration of exposure to the event increases, the probability of households using clean energy sources also increases. This suggests that longer exposure to the improved water and sanitation infrastructure has a more pronounced effect on the transition to clean energy in households. Based on this finding, the subsequent empirical analysis in this study constructs variables to measure the duration of individuals’ exposure to the renovation of tap water and sanitary toilets, in order to analyze the extent of its impact on clean energy adoption.

### 3.2. Methodology and empirical strategy

This study’s core identification strategy relies on exploiting the exogenous variation in the implementation of the renovation of tap water supply and sanitary toilets at the village level. First, the implementation of the rural tap water and sanitary toilet renovation project is taken as the core explanatory variable to evaluate its overall impact on household clean energy use, thereby verifying whether the policy significantly promotes clean energy adoption on average. Subsequently, the analysis incorporates the exposure duration of households after the policy implementation to examine the marginal effects of varying lengths of policy exposure on clean energy use, thus revealing the cumulative and dynamic characteristics of the policy effects over time.

To formally examine the influence of improved water and sanitation facilities on clean energy adoption, we specify the following equation:


$${Clean}_{ivt}=\alpha_{\text{0}}+\alpha_{\text{1}}{Renovate}_{vt}+X\alpha +\theta_{c}+\tau_{t}+\varepsilon_{ivt}$$ (1)

where Clean represents the types of energy (coal, firewood, natural gas, marsh gas, etc.) used by household *i* in village *v* at time *t,* as well as a dummy variable indicating whether the household uses clean energy. *Renovate* represents whether village *v* implemented the restructuring of tap water and sanitary toilets during time period *t*, which takes the value of 1 if village *v* has already implemented the project, and 0 otherwise. The coefficient of interest is denoted as $\alpha_{\text{1}}$, captures the effect of this reform on the adoption of clean energy in households. *X* represents a series of control variables, including personal characteristics such as the gender, age, and educational level of the household head, as well as household demographic and economic features such as household size, income level and asset status. θ represents the fixed effects for regions, τ represents the fixed effects at time *t*, and ε represents the error term. To account for the correlation of individuals’ unobservable characteristics within villages, standard errors are clustered at the village level.

To explore the impact of the duration of exposure to the renovation of tap water supply and sanitary toilets on household clean energy adoption, this paper employs a continuous treatment variable measuring the length of time individuals have been experienced the “improved water and sanitation” intervention. The treatment variations across samples arise from differences in individuals’ birth dates and the temporal disparities in the village-level implementation of the reform. The advantage of using this continuous treatment variable in the empirical analysis is that it allows for the full utilization of sample variation, thereby enhancing the efficiency of empirical identification. The specific identification model is represented by Equation (2) as follows:


$${Clean}_{ivt}=\beta_{\text{0}}+\beta_{\text{1}}{expose}_{ivt}+X\beta +\theta_{c}+\tau_{t}+\varepsilon_{ivt}$$ (2)

where ${\text{e}xpose}_{ivt}\;$denotes the duration of individuals’ exposure to the renovation of tap water and sanitary toilets. If the village has never implemented the reform, ${\text{e}xpose}_{ivt}$ is assigned a value of 0. In cases where the village has implemented the reform and the intervention took place after an individual’s birth, ${\text{e}xpose}_{ivt}$ is computed as the difference between the survey year and the year of reform implementation. Additionally, if the village implemented the reform before an individual’s birth, then ${\text{e}xpose}_{ivt}$ is equal to the individual’s age. Specifically, the calculation method for the duration of exposure to the reform, influenced by the reform, is shown in Equation (3).


$${Clean}_{ivt}=\alpha_{\text{0}}+\alpha_{\text{1}}{Renovate}_{vt}+X\alpha +\theta_{c}+\tau_{t}+\varepsilon_{ivt}$$ (3)

In addition, to investigate the potential heterogeneity in the effects of exposure duration on clean energy adoption, this study classified the continuous exposure variable into five groups: 0 years, 0–3 years, 3–5 years, 5–10 years, and 10 years or more. This grouping enables a more nuanced analysis of how varying lengths of exposure to the renovation of tap water and sanitary toilets may differentially influence household clean energy adoption. The corresponding estimation results are reported in S1 Table.

The validity of the empirical analysis relies on the exogeneity of the explanatory variables. In this paper, the explanatory variable is whether the village where the household is located has implemented the renovation of tap water and sanitary toilets. The implementation of this project and its specific process are entirely determined by the local government and village collective, independent of individuals or households. Therefore, the paper considers the identification and estimation based on the aforementioned strategy to be valid.

## 4. Empirical results

### 4.1. Main results

The main empirical results are presented in Table 3. Households are more likely to adopt clean energy after the renovation of tap water and sanitary toilets, which is consistent with the results in Table 2. Specifically, the renovation of tap water and sanitary toilets reduces the probability of households using coal and firewood for cooking by 3.3 and 4.5 percentage points, respectively, while increasing the probability of households using gas and clean energy for cooking by 6.9 and 8.0 percentage points, respectively. All estimated effects are statistically significant at the 1% level. Based on these results, Hypothesis 1 is confirmed.

**Table 3:** The effects of the renovation of tap water and sanitary toilets on household clean energy adoption.

Variables	(1)	(2)	(3)	(4)
	Coal	Firewood	Gas	Cleanenergy
Renovate	−0.033***	−0.045***	0.069***	0.080***
	(0.007) [-0.047, -0.019]	(0.012) [-0.068, -0.021]	(0.013) [0.043, 0.095]	(0.015) [0.051, 0.110]
Age	0.001	0.000	−0.000	−0.000
	(0.000) [-0.000, 0.001]	(0.000) [-0.001, 0.001]	(0.001) [-0.001, 0.001]	(0.000) [-0.001, 0.000]
Gender	−0.007**	0.049***	−0.013*	−0.049***
	(0.003) [-0.014, -0.000]	(0.014) [0.021, 0.077]	(0.007) [-0.026, 0.000]	(0.011) [-0.070, -0.027]
Marital status	0.014	0.109***	−0.065***	−0.116***
	(0.011) [-0.009, 0.036]	(0.018) [0.075, 0.144]	(0.018) [-0.100, -0.029]	(0.021) [0.158, -0.744]
Education	0.001	−0.016***	0.008***	0.015***
	(0.001) [-0.000, 0.003]	(0.001) [-0.018, -0.014]	(0.001) [0.006, 0.011]	(0.001) [0.013, 0.017]
Medical insurance	0.002	−0.031*	0.020	0.035***
	(0.020) [-0.037, 0.041]	(0.018) [-0.067, 0.004]	(0.015) [-0.010, 0.050]	(0.011) [0.013, 0.057]
Pension insurance	0.007	0.050***	−0.042***	−0.065***
	(0.005) [-0.003, 0.017]	(0.007) [0.038, 0.063]	(0.007) [-0.055, -0.028]	(0.006) [-0.077, -0.054]
Family size	0.001	0.004	0.001	−0.005
	(0.003) [-0.004, 0.005]	(0.006) [-0.009, 0.016]	(0.003) [-0.004, 0.007]	(0.007) [-0.019, 0.009]
CDR	0.005	0.026	−0.010	−0.019
	(0.011) [-0.015, 0.026]	(0.034) [-0.040, 0.091]	(0.016) [-0.040, 0.020]	(0.035) [-0.089, 0.050]
ODR	0.007	0.067***	−0.052***	−0.070***
	(0.148) [-0.020, 0.033]	(0.008) [0.051, 0.083]	(0.015) [-0.081, -0.023]	(0.009) [-0.087, -0.053]
Lnincome	−0.007***	−0.051***	0.048***	0.057***
	(0.002) [-0.011, -0.004]	(0.010) [-0.070, -0.031]	(0.008) [0.033, 0.063]	(0.010) [0.038, 0.076]
Lnassets	−0.004***	−0.015***	0.016***	0.019***
	(0.002) [-0.007, -0.001]	(0.002) [-0.019, -0.011]	(0.002) [0.011, 0.020]	(0.002) [0.015, 0.022]
Provincial FE	YES	YES	YES	YES
Time FE	YES	YES	YES	YES
N	8736	9585	9684	9585

Note: Marginal effects are reported in the probit model. Clustered robust standard errors at the village level are presented in parentheses. The values in square brackets represent the 95% confidence intervals. *p < 0.10; **p < 0.05; ***p < 0.01.

For the other control variables, the estimated results are consistent with our expectations and the findings of previous studies. Specifically, the proportion of elderly individuals within a household exerts a significant influence on household energy consumption patterns. A higher elderly population share is associated with a decreased probability of clean energy adoption, a statistically significant increase in the use of firewood, and an elevated but statistically insignificant likelihood of coal usage. These findings imply that households with a greater concentration of elderly members tend to rely more heavily on traditional solid fuels. This may be because elderly individuals tend to have a weaker ability to adapt to new technologies and are more accustomed to using traditional solid fuels [42], thereby reducing the adoption of clean energy in households as their proportion increases.

Table 3 demonstrates that married households are more inclined to use firewood. A possible explanation is that married households often have larger family sizes and greater cooking demands, leading them to prefer for high-calorific, cost-effective fuels such as firewood [43]. In contrast, unmarried or single-person households typically have simpler lifestyles and smaller cooking needs, making the adoption of smaller-scale, cleaner cooking technologies more feasible.

Education enhances people’s understanding and awareness of environmental issues, fostering stronger environmental consciousness, greener attitudes, and a sustainable development mindset [44]. Consequently, individuals with higher education levels are more likely to engage in green consumption behaviors and environmental actions [45], thereby increasing the likelihood of adopting clean energy sources. Additionally, higher household income and wealth can enhance both the affordability and willingness of respondents to use clean energy [16,46], thus facilitating the household energy transition.

To further investigate the impact of the duration of the renovation of tap water and sanitary toilets on household clean energy adoption, Table 4 presents the estimated results based on Equation (3). The results show that as the renovation duration increases, the probability of households using traditional energy sources (such as coal and firewood) for cooking decreases, while the probability of using clean energy sources (such as electricity, natural gas, marsh gas, and liquefied petroleum gas) increases. Specifically, for each additional year of renovation duration, the probability of households using coal and firewood for cooking decreases by 0.2 and 0.3 percentage points, respectively, while the probability of using gas and clean energy sources for cooking increases by 0.4 and 0.5 percentage points, respectively.

**Table 4:** The effects of the duration of the renovation of tap water and sanitary toilets on household clean energy adoption.

Variables	(1)	(2)	(3)	(4)
	Coal	Firewood	Gas	Cleanenergy
Expose duration	−0.002***	−0.003***	0.004***	0.005***
	(0.001)	(0.001)	(0.001)	(0.001)
Age	0.001	0.000	−0.000	−0.000
	(0.000)	(0.000)	(0.000)	(0.000)
Gender	−0.007*	0.049***	−0.012*	−0.049***
	(0.004)	(0.014)	(0.007)	(0.011)
Marital status	0.014	0.109***	−0.064***	−0.116***
	(0.011)	(0.017)	(0.017)	(0.021)
Education	0.001	−0.016***	0.008***	0.015***
	(0.001)	(0.001)	(0.001)	(0.001)
Medical insurance	0.003	−0.030*	0.018	0.033***
	(0.019)	(0.018)	(0.016)	(0.011)
Pension insurance	0.007	0.050***	−0.042***	−0.065***
	(0.005)	(0.006)	(0.007)	(0.006)
Family size	0.000	0.004	0.001	−0.005
	(0.002)	(0.007)	(0.003)	(0.007)
CDR	0.009	0.025	−0.007	−0.018
	(0.011)	(0.034)	(0.016)	(0.036)
ODR	0.007	0.068***	−0.053***	−0.071***
	(0.013)	(0.008)	(0.015)	(0.008)
Lnincome	−0.007***	−0.051***	0.049***	0.057***
	(0.002)	(0.010)	(0.007)	(0.010)
Lnassets	−0.004***	−0.015***	0.016***	0.019***
	(0.002)	(0.002)	(0.002)	(0.002)
Provincial FE	YES	YES	YES	YES
Time FE	YES	YES	YES	YES
N	8736	9585	9684	9585

Note: Marginal effects are reported in the Probit model. Clustered robust standard errors at the village level are presented in parentheses. *p < 0.10; **p < 0.05; ***p < 0.01.

When the duration of the renovation of tap water and sanitary toilets is treated as a categorical variable, the estimated results are presented in S1 Table. The empirical results also indicate that as the duration of the renovation increases, the impact of the renovation on household clean energy adoption becomes stronger. Specifically, for clean energy adoption, the estimated coefficients for duration categories of 0–3 years, 3–5 years, 5–10 years, and over 10 years are 0.135, 0.163, 0.086, and 0.068, respectively. Furthermore, longer durations correspond with greater statistically significance of the coefficients, providing additional support for Hypothesis 2.

Previous empirical results in this study have shown that the renovation of tap water and sanitary toilets can increase clean energy usage in rural households. However, there are differences in residents’ general acceptance of “improving access to tap water” versus “sanitary toilet renovation”, and significant cost differences exist between the two interventions. In many cases, “improving access to tap water” is a basic prerequisite for “sanitary toilet renovation”. Based on this, we next attempt to separately evaluate the long-term effects of each intervention on clean energy adoption. In the CHARLS 2014 Life History Survey, respondents were asked about their experiences with using tap water and having access to sanitary toilets since birth. In this study, we constructed binary variables indicating whether individuals had access to tap water and toilets in their childhood households. These variables are simultaneously included in Equation (1). Table 5 presents the differential effects of “improving tap water” and “sanitary toilet renovation” on clean energy adoption. Specifically, access to clean water in respondents’ households during childhood increases the probability of household clean energy use by 5.5 percentage points. Similarly, availability of water toilets in respondents’ households during childhood increases this probability by 13.5 percentage points. By comparing the coefficients of “Clean water” and “Water closet” in columns (2)-(4) of Table 5, it can be observed that the effect of household sanitary toilet improvement on increasing clean energy use probability is significantly larger than that of tap water improvement. This indicates that the renovation of sanitary toilets in rural areas has a greater impact on facilitating household energy transition.

**Table 5:** The differential effects of improving tap water and sanitary toilet on clean energy adoption.

Variables	(1)	(2)	(3)	(4)
	Coal	Firewood	Gas	Cleanenergy
Clean water	−0.019**	−0.024**	0.033***	0.055***
	(0.008)	(0.009)	(0.010)	(0.010)
Sanitary toilets	0.001	−0.142***	0.117***	0.135***
	(0.013)	(0.043)	(0.019)	(0.046)
Age	0.001	0.001	−0.001	−0.001
	(0.001)	(0.000)	(0.001)	(0.001)
Gender	−0.023***	0.055**	−0.031	−0.039**
	(0.007)	(0.022)	(0.022)	(0.017)
Marital status	0.001	0.103***	−0.051***	−0.098***
	(0.011)	(0.027)	(0.005)	(0.013)
Education	0.001	−0.014***	0.008***	0.013***
	(0.001)	(0.001)	(0.001)	(0.001)
Medical insurance	0.043**	−0.040***	0.015	0.010
	(0.018)	(0.014)	(0.075)	(0.011)
Pension insurance	0.017**	0.028	−0.025	−0.045**
	(0.007)	(0.022)	(0.019)	(0.021)
Family size	0.004	0.003	0.002	−0.005
	(0.005)	(0.004)	(0.001)	(0.003)
CDR	0.032	−0.070	−0.010	0.042
	(0.031)	(0.076)	(0.047)	(0.103)
ODR	−0.014**	0.065	−0.010	−0.056
	(0.006)	(0.049)	(0.046)	(0.058)
Lnincome	−0.007***	−0.016	0.013***	0.020*
	(0.002)	(0.014)	(0.004)	(0.011)
Lnassets	0.004**	−0.039**	0.021*	0.036*
	(0.002)	(0.018)	(0.011)	(0.020)
Provincial FE	YES	YES	YES	YES
Time FE	YES	YES	YES	YES
N	5400	6051	5942	6044

Note: If the respondents had access to clean tap water during their childhood, the variable “water” is assigned a value of 1; otherwise, it is assigned a value of 0. Similarly, if the respondents had access to water closet during their childhood, the variable “closet” is assigned a value of 1; otherwise, it is assigned a value of 0. Marginal effects are reported in the probit model. Clustered robust standard errors at the village level are presented in parentheses. *p < 0.10; **p < 0.05; ***p < 0.01.

This study further examines the relationship between the duration of tap water and sanitary toilets renovations and household clean energy adoption using data from the CHARLS 2014 Life History Survey. The main results are reported in S2 Table and are generally consistent with the previous analysis.

Additionally, this study does not use household-level “tap water and sanitary toilets renovation” history as a baseline. This is because household adoption of tap water or sanitary toilets is clearly not randomly assigned and is subject to confounding by unobservable factors such as household characteristics, which can lead to severse selection bias. By focusing on the village-level analysis, we can mitigate endogeneity concerns caused by spillover effects and self-selection biases, enabling a more reliable assessment of the overall impact of tap water and sanitary toilet renovations.

### 4.2 Robustness checks

(1) Propensity score matching

There may be systematic differences between the villages that participated in the renovation of tap water and sanitary toilets and those that did not, leading to non-random adoption of the renovation across villages. To address this concern, this study uses propensity score matching for robustness analysis, which helps mitigate potential selection bias and non-randomness, thus enhancing comparability between reform and non-reform villages.

Specifically, three matching methods—1:3 nearest neighbor matching, kernel matching, and radius matching— are used to calculate the propensity scores for each village regarding the “improved tap water and sanitary toilets” intervention, based on a logit model. Due to the unavailability of pre-intervention village characteristics, we can only calculate the propensity scores for each village based on the village characteristics observed in 2011. In the estimation process of propensity scores, the matching variables include the main topography and geomorphology of the village, weather conditions at the village location, road types in the village, whether there are large surnames in the village, whether the village has a sewage system, the total population residing in the village for more than half a year, the proportion of the population with at least a middle school education in the village, the number of individuals in the village who attended university in the past five years, and the per capita disposable income of the village. These aforementioned factors are not only related to the prosperity level of the village but also correlated with its collective action capability. This can influence whether the village implements the renovation of tap water and sanitary toilets. The balance test results for the three propensity score matching methods are reported in S3 Table in the Supporting information. After matching, the between-group differences for all variables are not significant at the 10% level, indicating that the matching is effective.

Table 6 presents the effects of tap water and sanitary toilets improvement on clean energy adoption under different matching methods. Across all three matching methods, the effects of the renovation on the probability of households using coal, firewood, gas, and clean energy are statistically significant at the 1% level, and the coefficients are consistent with those in Table 3. The robustness results further confirm that the renovation contributes to promoting the transition of rural households towards clean energy usage.

**Table 6:** Robustness test: propensity score matching.

Matching type	(1)	(2)	(3)	(4)
	Coal	Firewood	Gas	Cleanenergy
1:3 nearest neighbor matching	−0.039***	−0.031***	0.070***	0.072***
	(0.008)	(0.011)	(0.013)	(0.012)
kernel matching	−0.032***	−0.046***	0.070***	0.080***
	(0.007)	(0.012)	(0.013)	(0.013)
radius matching	−0.041***	−0.036***	0.073***	0.077***
	(0.007)	(0.011)	(0.013)	(0.012)

Note: Marginal effects are reported in the Probit model. The control variables include age, gender, marital status, education, medical insurance, pension insurance, family size, CDR, ODR, Lnincome, Lnassets, Province fixed effects and time fixed effects. Clustered robust standard errors at the village level are presented in parentheses. *p < 0.10; **p < 0.05; ***p < 0.01.

(2) Eliminate the influence of confounding factors

In rural areas of China, while promoting the renovation of tap water and sanitary toilets, the government has also implemented many other significant policies to drive rural economic development. These reforms may be correlated with the renovation of tap water and sanitary toilets and may also influence household clean energy usage, leading to interference in assessing the effects of the improvement of tap water and sanitary toilets. As a result, we aim to control for these confounding factors separately and re-evaluate the effect of this policy.

Firstly, due to the rapid decline in fertility rates and the accelerated pace of urbanization, the demand for education in rural areas of China has decreased, leading to an inefficient allocation of educational resources. To improve the efficiency of rural education, China formally initiated the consolidation of schools in some rural areas in 2001, resulting in the closure of certain schools. The consolidation of rural schools can lead to a reduction of 0.6 years in the educational attainment of rural children [47]. This decline in educational attainment may influence households’ awareness and usage of clean energy. Therefore, it is necessary to further control for the impact of school consolidation on clean energy adoption. A dummy variable is constructed to indicate whether the household’s village underwent school consolidation before 2011. After controlling for this variable, the estimated results are reported in the third row of Table 7, which are generally consistent with the empirical results in Table 3.

**Table 7:** Robustness test: Eliminate the influence of confounding factors.

Confounding factors	(1)	(2)	(3)	(4)
	Coal	Firewood	Gas	Cleanenergy
The primary schools merged or dissolved	−0.036***	−0.050***	0.072***	0.088***
	(0.007)	(0.012)	(0.014)	(0.015)
Control the factors affecting educational supply	−0.033***	−0.040***	0.065***	0.076***
	(0.007)	(0.011)	(0.013)	(0.014)
Land adjustments after the renovation of tap water and sanitary toilets	−0.029***	−0.034***	0.064***	0.071***
	(0.007)	(0.011)	(0.011)	(0.012)
All three factors are controlled	−0.030***	−0.036***	0.065***	0.074***
	(0.007)	(0.010)	(0.011)	(0.011)

Note: Marginal effects are reported in the probit model. The values in the table indicate the impact of the renovation of tap water and sanitary toilets on household clean energy usage after further controlling for confounding factors such as school consolidation, education supply, and land adjustment, in addition to the variables already controlled for in Table 4. Clustered robust standard errors at the village level are presented in parentheses. *p < 0.10; **p < 0.05; ***p < 0.01. The number of observations for the confounding factors are 9,657 for school consolidation, 9,684 for education supply, and 9,684 for land adjustment.

Secondly, the influence of village-level educational resources on household clean energy usage is excluded. Basic education has been vigorously promoted in rural China, and better the local educational resources are generally associated with greater environmental awareness among residents. Based on the village questionnaire in 2011, the number of individuals from the households’ village who enrolled in university over the past five years is used as a proxy variable for the local supply of educational resources. After controlling for this variable, the estimated results in Table 7 continue to indicate that the probability of households using clean energy increases following the renovation of tap water and sanitary toilets, with only minimal difference from the baseline regression results.

Thirdly, land is a crucial form of capital and a fundamental safeguard for rural household production. Changes in land circumstances may impact rural household income and in turn, affect the affordability of clean energy adoption. In the robustness analysis, we control for the variable of whether the land in the village has been adjusted after the renovation of tap water and sanitary toilet. After controlling for the variable of whether there has been a change in land resources, the results continue to support a positive association between the renovation of tap water and sanitary toilets and household clean energy adoption.

Finally, when simultaneously controlling for the all three confounding factors discussed above, the estimated results, as shown in the last row of Table 7, still indicate a significant positive effect of the renovation of tap water and sanitary toilets on clean energy usage, conforming the robustness of the results.

### 4.3. Heterogeneity analysis

Thus far, we have empirically investigated the heterogeneity of the impact of this renovation on household clean energy adoption from two perspectives: the gender of the household head and household income.

In developing countries, women often place a higher value on access to clean water sources and sanitary toilets compared with men. Previous studies have confirmed that the benefits of tap water and sanitary toilet renovation tend to be greater for women than for men [26]. The use of dirty fuels or contaminated water resources imposes a disproportionate health burden on women [10]. This raises the question of whether there is a gender difference in the impact of tap water and sanitary toilet renovation on clean energy adoption. To address this, we examine the heterogeneity by gender through the interaction between tap water and sanitary toilet renovation and head gender.

The results, presented in Table 8, show that for traditional energy sources such as coal and firewood, the coefficients of the interaction term are negative and statistically significant (As shown in columns (1) and (2) of Table 8). In contrast, for clean energy, the coefficients of the interaction term are positive (As shown in (3) and (4) of Table 8). More specifically, the results reveal that, compared with households headed by female, the renovation of tap water and sanitary toilets is associated with a 4.1-percentage-point increase in the probability of clean energy adoption for households headed by male heads. In general, females tend to have stronger environmental awareness and a greater inclination toward using clean energy compared than males. This assertion is also supported by the baseline results in Table 3, which indicates that households with female heads have a 4.9-percentage-point higher probability of using clean energy compared with households with male heads. However, our estimation results indicate that under the influence of the tap water and sanitary toilet renovation policy, male-headed households also exhibit an increased inclination toward clean energy usage. The renovation of tap water and sanitary toilets appears to have reduced the gender disparity in terms of clean energy adoption.

**Table 8:** Heterogeneity effects of gender.

Variables	(1)	(2)	(3)	(4)
	Coal	Firewood	Gas	Cleanenergy
Renovate*Gender	−0.026***	−0.021**	0.016	0.041***
	(0.008)	(0.010)	(0.013)	(0.008)
Renovate	−0.021**	−0.034***	0.061***	0.060***
	(0.008)	(0.013)	(0.013)	(0.016)
Gender	0.001	0.058***	−0.021***	−0.066***
	(0.005)	(0.014)	(0.007)	(0.011)
Other variables	YES	YES	YES	YES
Provincial FE	YES	YES	YES	YES
Time FE	YES	YES	YES	YES
N	8736	9585	9684	9585

Note: When gender equals 1, it indicates male. Marginal effects are reported in the probit model. The other variables include age, gender, marital status, education, medical insurance, pension insurance, family size, CDR, ODR, Lnincome and Lnassets. Clustered robust standard errors at the village level are presented in parentheses. *p < 0.10; **p < 0.05; ***p < 0.01.

Normally, higher-income households typically have a greater capacity to afford clean energy adoption, and the use of clean energy tends to increase as household income rises. This trend is also observed in the baseline regression analysis, as shown in column (4) of Table 3. With a 1% increase in income, the probability of households using clean energy is expected to increase by 5.7 percentage points. Therefore, under the influence of the renovation of tap water and sanitary toilets, is there heterogeneity with respect to income? In other words, does the tendency of household clean energy adoption to rise with income persist when the renovation of tap water and sanitary toilets policy is in place? To investigate this, we divided households into three income groups at the provincial level: high-income, middle-income, and low-income households. The estimation results for income heterogeneity are presented in Table 9.

**Table 9:** Heterogeneity effects of income.

Variables	(1)	(2)	(3)	(4)
	Coal	Firewood	Gas	Cleanenergy
Renovate* Low income	−0.031***	−0.059***	0.088***	0.098***
	(0.005)	(0.015)	(0.013)	(0.018)
Renovate* Middle income	−0.033***	−0.041***	0.071***	0.075***
	(0.011)	(0.012)	(0.016)	(0.014)
Renovate* High income	−0.033***	−0.030**	0.049***	0.065***
	(0.009)	(0.011)	(0.012)	(0.015)
Low income	0.023**	0.058**	−0.062**	−0.070***
	(0.011)	(0.024)	(0.025)	(0.017)
Middle income	0.008	0.070***	−0.046***	−0.067***
	(0.007)	(0.010)	(0.013)	(0.008)
Other variables	YES	YES	YES	YES
Provincial FE	YES	YES	YES	YES
Time FE	YES	YES	YES	YES
N	8736	9585	9684	9585

Note: Marginal effects are reported in the probit model. The other variables include age, gender, marital status, education, medical insurance, pension insurance, family size, CDR, ODR, Lnincome and Lnassets. Clustered robust standard errors at the village level are presented in parentheses. *p < 0.10; **p < 0.05; ***p < 0.01.

As indicated in Table 9, the interference of the renovation of tap water and sanitary toilets suggests a stronger inclination toward clean energy usage among low-income households. For example, in column (4) in Table 9, the coefficients of the interaction terms between the renovation policy (*Renovate*) and the low-, middle- and high-income groups are 0.098, 0.075, and 0.065, respectively, all significant at the 1% level. These estimation results indicate that the renovation of tap water and sanitary toilets can alter the propensity of low-income households to adopt clean energy. After the policy’s implementation, the adoption of clean energy in households is no longer determined solely by affordability.

### 4.4 Potential mechanisms

In this section, we investigate the underlying mechanisms through which the renovation of tap water and sanitary toilets promotes household adoption of clean energy, focusing on three channels: education level, cognitive abilities, and environmental awareness. Our analysis is guided by the hypotheses outlined in Section 2.

The education mechanism is documented in Table 10. Before conducting empirical testing, it is important to note that respondents in the CHARLS sample are all aged 45 and above, most of whom were already adults when the renovation of tap water and sanitary toilets policy was implemented. By that time, their formal education had largely concluded. This may limit the applicability of using the CHARLS data to directly examine the policy’s impact on respondents’ own educational levels. Fortunately, the CHARLS has consistently colllected information on respondents’ children in various rounds, including gender, birth year, and educational history. These children may have benefitted from the improved access to clean tap water and sanitary toilets since their childhood or adolescence as the result of the policy changes, which could in turn influence their educational attainment. We therefore focus on a subgroup of younger children of the respondents. Based on the available data, we select children born between 1970 and 1997 for analysis. This ensures that, by the survey year, these individuals have already completed basic compulsory education and that their years of schooling were not influenced by subsequent extensions to mandatory schooling requirements.

**Table 10:** Effects of the renovation of tap water and sanitary toilets on education.

Variables	(1)	(2)	(3)	(4)	(5)	(6)
	Years of education		Completed high school or not		Attending university or not.	
Renovate	0.006*		0.027***		0.010***	
	(0.003)		(0.007)		(0.003)	
Expose duration		0.000		0.002***		0.001*
		(0.000)		(0.001)		(0.000)
Gender of children	0.024**	0.024**	0.009***	0.010***	−0.001	−0.001
	(0.010)	(0.010)	(0.004)	(0.003)	(0.003)	(0.003)
Number of children	−0.008***	−0.008***	−0.029***	−0.029***	−0.019***	−0.019***
	(0.001)	(0.001)	(0.001)	(0.001)	(0.002)	(0.002)
Average years of education for parents	0.011***	0.011***	0.032***	0.032***	0.019***	0.019***
	(0.001)	(0.001)	(0.001)	(0.001)	(0.001)	(0.001)
Whether to merge schools or not	0.013***	0.013***	−0.004	−0.004	−0.003	−0.003
	(0.004)	(0.004)	(0.005)	(0.005)	(0.004)	(0.004)
Number of villagers admitted to college	0.000**	0.000**	0.001***	0.001***	0.000***	0.000***
	(0.000)	(0.000)	(0.000)	(0.000)	(0.000)	(0.000)
Whether land has been adjusted or not	0.014***	0.014***	0.019***	0.015*	−0.009**	−0.011**
	(0.004)	(0.004)	(0.007)	(0.008)	(0.004)	(0.005)
Occurred major natural disasters or not in the past five years	0.005	0.005	−0.013***	−0.014***	−0.014***	−0.014***
	(0.003)	(0.003)	(0.004)	(0.004)	(0.003)	(0.003)
Fixed effects for birth year	YES	YES	YES	YES	YES	YES
N	20709	20709	20709	20709	20709	20709

Note: Clustered robust standard errors at the village level are presented in parentheses. *p < 0.10; **p < 0.05; ***p < 0.01.

The dependent variables include the education years of the interviewed respondents’ children, whether they have completed high school, and whether they have attended college. Considering the results from Table 10, it can be observed that, following the implementation of tap water and sanitary toilet renovation, the education years of the respondents’ children increased approximately by 0.006 years. The probability of completing high school increased significantly by 2.7 percentage points at the 1% significance level, while the probability of attending college has risen by 1 percentage point at the same significance level. Similarly, it can be observed that the longer the duration of exposure to tap water and sanitary toilet renovation, the greater the increase in educational attainment.

The renovation of tap water and sanitary toilets can improve individual health and enhance cognitive abilities [48]. Changes in cognitive abilities can alter individuals’ acceptance and awareness of novel concepts, such as clean energy. Columns (1) and (2) of Table 11 present the impact of early experiences with improved water and sanitation facilities on cognitive abilities, aiming to explore the mechanisms through which such improvements influence households’ adoption of clean energy.

**Table 11:** Effects of the renovation of tap water and sanitary toilets on cognitive ability and environmental awareness.

Variables	(1)	(2)	(3)	(4)	(5)	(6)
	Cognitive ability		Indoor cleanliness level		Indoor cleanliness or not	
Renovate	0.195***		0.128***		0.020***	
	(0.067)		(0.044)		(0.006)	
Expose duration		0.015***		0.004		0.001**
		(0.004)		(0.003)		(0.000)
Other variables	YES	YES	YES	YES	YES	YES
Provincial FE	YES	YES	YES	YES	YES	YES
Time FE	YES	YES	YES	YES	YES	YES
N	9684	9684	8212	8212	9684	9684

Note: Marginal effects are reported in the probit model. The other variables include age, gender, marital status, education, medical insurance, pension insurance, family size, CDR, ODR, Lnincome and Lnassets. Clustered robust standard errors at the village level are presented in parentheses. *p < 0.10; **p < 0.05; ***p < 0.01.

The key to identifying the impact of improved water and sanitation facilities lies in determining effective indicators for measuring cognitive abilities in middle-aged and elderly individuals. The Mini-Mental State Examination (MMSE) is commonly used to assess dementia and the degree of cognitive impairment. It generally includes assessments of time orientation, spatial orientation, short-term memory, delayed memory, attention, calculation ability, language, and visuospatial skills. The CHARLS dataset from 2011 to 2018 includes only a subset of questions from the MMSE scale, primarily focusing on aspects of time orientation, memory, and calculation ability. Time orientation assesses the respondent’s awareness of the year, month, day, season, and day of the week. Memory ability primarily examines the respondent’s short-term and delayed memory capabilities. During the survey, interviewers present a list of ten words to the respondent and then ask them to recall the words at two different time points. Calculation ability is evaluated by asking the respondent subtract seven from 100 successively for five times. The simplified method used in this study to calculate cognitive test scores involves a total of 30 questions. Each correct answer is awarded one point, and a higher score indicates better cognitive function.

The results in columns (1) and (2) of Table 11 indicate that, after experiencing improvements in water and sanitation facilities, respondents’ cognitive abilities increase significantly by 0.195 at the 1% level of significance. Additionally, for each year of exposure to these improvements, cognitive abilities increase by 0.015. The earlier hypothesis that improvements in water and sanitation promote the household energy transition by enhancing the cognitive abilities of household heads, has been validated through the empirical results.

Values such as green awareness, green attitudes, environmental concern, and trust all have an impact on residents’ green consumption and environmentally friendly behaviors [16,49]. Residents with higher environmental awareness often integrate environmental values into their lifestyles. They may be more willing to choose energy-efficient products to reduce energy consumption and environmental pollution, thereby minimizing their environmental impact [50,51]. Taking data availability into account, we use the cleanliness level of the indoor household environment as a proxy variable for environmental awareness. The CHARLS includes responses regarding the indoor environmental cleanliness of respondents’ living environments. The higher the level of cleanliness, the stronger we assume their environmental awareness to be. Additionally, we constructed a dummy variable for environmental awareness, assigning a value of 1 when indoor cleanliness is rated as “Excellent,” “Very clear,” or “Clear,” indicating a very strong environmental awareness, and 0 otherwise. The estimated results of the impact of tap water and sanitary toilet renovations on environmental awareness are reported in columns (3) to (6) of Table 11. Consistent with our expectations, the renovation significantly increased household environmental awareness. The earlier hypothesis, that the renovation of tap water and sanitary toilets facilitates a transition in household energy usage through the enhancement of homeowners’ environmental awareness, has been confirmed.

## 5. Conclusions and implications

The rural water and toilet improvement project is an essential component of China’s recent rural infrastructure development, serving as a key measure to enhance the living environment and quality of life for rural residents. Nevertheless, the impact of this project on residents’ lifestyles and the household energy transition has not yet been clearly, systematically, or quantitatively examined. This research makes a significant contribution by addressing this gap, assessing the impact of the rural water and toilet improvement project on household clean energy adoption using CHARLS data from 2011 to 2018. In addition to analyzing the entire sample, this study further investigates heterogeneity in clean energy adoption among different demographic groups and explores the underlying mechanisms. By deepening our understanding of the relationship between water and toilet improvements and the household energy transition, this study offers valuable insights that can contribute to the social benefits of improving human habitat environment in both China and other developing countries.

The main findings are as follows. The renovation of tap water and sanitary toilets has a significant positive impact on promoting the transition to cleaner energy sources in rural households. Specifically, the implementation of improved water and toilet facilities reduces the probability of rural households using coal and firewood for cooking by 3.3 and 4.5 percentage points, respectively, while increasing the probability of using gas and clean energy by 6.9 and 8.0 percentage points. Furthermore, this renovation has a greater impact on the transition to clean energy for households with male heads and lower income levels. Improvements in education and cognitive capabilities contribute to human capital accumulation and the strengthening of environmental awareness, which are crucial pathways through which the renovation encourages households to adopt clean energy more extensively.

This study holds significant policy implications for improving water and public sanitation in developing countries, as well as promoting the transition towards clean energy usage. Firstly, improving water supply and sanitation facilities can facilitate rural energy transformation and improve living conditions, which is crucial for residents’ well-being. Therefore, there is a need to strengthen investment in water security and public sanitation facilities in rural areas of China, and the findings may also be relevant to regions in other developing countries facing similar rural infrastructure gaps. In addition, mechanism analysis reveals that improving water and toilets can promote the transition to clean energy by enhancing human capital, improving cognitive abilities, and raising environmental awareness. Given the significant spillover effects of clean energy adoption and human capital enhancement, the renovation of tap water and sanitary toilets can be seen as generating positive intertemporal externalities. Hence, active government efforts to strengthen rural drinking water and sanitation facilities can yield substantial societal benefits.

Secondly, improvement in living conditions can lead to greater adoption of clean energy and pro-environmental behaviors among men. From a gender perspective, it is notable that men generally have lower environmental and clean energy awareness compared to women. Nevertheless, this research reveals that improvements in water and sanitation have a stronger positive impact on male household heads, thereby altering this tendency. Improving water and sanitation facilities provides households with a clean, organized, and hygienic environment. Men can derive numerous benefits from such living environment, which may alter their perceptions and attitudes towards cleanliness. Consequently, they may become more inclined to adopt environmentally friendly and clean energy sources in their energy consumption. Hence, the continuous enhancement and refinement of the residential living environment can significantly enhance men’s environmental awareness and preferences, thereby further stimulating their pro-environmental behaviors.

Thirdly, it is necessary to further emphasize the impact of residential environment modification on clean energy utilization and environmentally friendly behaviors in low-income households. Low-income households often exhibit lower willingness and capacity to adopt high-cost clean energy due to limited affordability and financial capability. Nevertheless, this study reveals that the effect of improving water and sanitation facilities on promoting clean energy transition is more pronounced among low-income households. When living conditions related to water usage and sanitation are improved for these households, they are more likely to become conscious of environmental issues and make choices aligned with sustainable practices. This can create a positive feedback loop in which improved living conditions contribute to heightened environmental awareness, which in turn drives more pro-environmental behavior. Based on this, further improving and optimizing the living environment of low-income households can significantly enhance their environmental awareness and preferences, thereby further promoting their pro-environmental behaviors.

## Supporting information

S1 Table The effects of the duration of the renovation of tap water and sanitary toilets on household clean energy adoption. (DOCX)

S2 Table The duration of using tap water and water toilets in households and its impact on clean energy adoption. (DOCX)

S3 Table The balance test of propensity score matching. (DOCX)
