# Supplementary material for: Public sanitation interventions and household clean energy adoption: Evidence from China’s renovating water supply and toilets
Source: PLoS One. 2025 Oct 6;20(10):e0333630. doi: 10.1371/journal.pone.0333630 (PMC12500098; doi:10.1371/journal.pone.0333630)
Supplement: S1 Table — (DOCX) [file pone.0333630.s001.docx]

**S1 Table. The effects of the duration of the renovation of tap water and sanitary toilets on household clean energy adoption**

| Variables | (1) | (2) | (3) | (4) |
| --- | --- | --- | --- | --- |
|  | Coal | Firewood | Gas | Cleanenergy |
| expose3 | -0.013 | -0.115*** | 0.091*** | 0.135** |
|  | (0.016) | (0.038) | (0.023) | (0.040) |
| expose5 | -0.038* | -0.097 | 0.089* | 0.163** |
|  | (0.021) | (0.070) | (0.050) | (0.064) |
| expose10 | -0.042*** | -0.041** | 0.060*** | 0.086*** |
|  | (0.010) | (0.017) | (0.011) | (0.016) |
| expose11 | -0.021*** | -0.049*** | 0.068*** | 0.068*** |
|  | (0.006) | (0.012) | (0.016) | (0.013) |
| Other variables | YES | YES | YES | YES |
| Provincial FE | YES | YES | YES | YES |
| Time FE | YES | YES | YES | YES |
| N | 8736 | 9585 | 9684 | 9585 |

Note: Expose3, expose5, expose10, and expose11 represent the durations of experiencing water and toilet improvement for 3 years, 3-5 years, 5-10 years, and more than 10 years, respectively. Among these, the reference group is those with 0 years of experiencing water and toilet improvement. Marginal effects are reported in the probit model. The other variables include age, gender, marital status, education, medical insurance, pension insurance, family size, CDR, ODR, Lnincome and Lnassets. Clustered robust standard errors at the village level are presented in parentheses. *p < 0.10; **p < 0.05; ***p < 0.01.
