# Supplementary material for: Public sanitation interventions and household clean energy adoption: Evidence from China’s renovating water supply and toilets
Source: PLoS One. 2025 Oct 6;20(10):e0333630. doi: 10.1371/journal.pone.0333630 (PMC12500098; doi:10.1371/journal.pone.0333630)
Supplement: S2 Table — (DOCX) [file pone.0333630.s002.docx]

**S2 Table. The duration of using tap water and water toilets in households and its impact on clean energy adoption**

| Variables | (1) | (2) | (3) | (4) |
| --- | --- | --- | --- | --- |
|  | Coal | Firewood | Gas | Cleanenergy |
| Expose_water | 0.000 | -0.003*** | 0.002*** | 0.003*** |
|  | (0.000) | (0.001) | (0.001) | (0.001) |
| Expose_closet | 0.000 | -0.003*** | 0.001 | 0.003** |
|  | (0.001) | (0.001) | (0.001) | (0.001) |
| Other variables | YES | YES | YES | YES |
| Provincial FE | YES | YES | YES | YES |
| Time FE | YES | YES | YES | YES |
| N | 5737 | 6042 | 5933 | 6036 |

Note: “Expose_water” represents the duration (in years) since the respondent's household started using clean tap water. “Expose_closet” represents the duration (in years) since the respondent's household started using water closet. Marginal effects are reported in the probit model. The other variables include age, gender, marital status, education, medical insurance, pension insurance, family size, CDR, ODR, Lnincome and Lnassets. Clustered robust standard errors at the village level are presented in parentheses. *p < 0.10; **p < 0.05; ***p < 0.01.
