# Supplementary material for: Public sanitation interventions and household clean energy adoption: Evidence from China’s renovating water supply and toilets
Source: PLoS One. 2025 Oct 6;20(10):e0333630. doi: 10.1371/journal.pone.0333630 (PMC12500098; doi:10.1371/journal.pone.0333630)
Supplement: S3 Table — (DOCX) [file pone.0333630.s003.docx]

**S3 Table. The balance test of** **propensity score matching**

| Variables | Matching type | 1:3 nearest neighbor matching | | kernel matching | | radius matching | |
| --- | --- | --- | --- | --- | --- | --- | --- |
|  |  | t | p>\|t\| | t | p>\|t\| | t | p>\|t\| |
| Topography and geomorphology | Unmatched | -0.670 | 0.501 | -0.670 | 0.501 | -0.670 | 0.501 |
|  | Matched | 0.520 | 0.602 | 0.440 | 0.663 | 0.530 | 0.597 |
| Weather conditions | Unmatched | 0.890 | 0.372 | 0.890 | 0.372 | 0.890 | 0.372 |
|  | Matched | 0.790 | 0.433 | 0.380 | 0.701 | 0.770 | 0.443 |
| Road types | Unmatched | 2.910 | 0.004 | 2.910 | 0.004 | 2.910 | 0.004 |
|  | Matched | -0.490 | 0.622 | 0.030 | 0.978 | -0.380 | 0.705 |
| Large surnames | Unmatched | -0.210 | 0.832 | -0.210 | 0.832 | -0.210 | 0.832 |
|  | Matched | 0.790 | 0.430 | 0.560 | 0.573 | 0.830 | 0.409 |
| Sewage system | Unmatched | 4.750 | 0.000 | 4.750 | 0.000 | 4.750 | 0.000 |
|  | Matched | -0.300 | 0.768 | -0.410 | 0.680 | -0.300 | 0.768 |
| Total population | Unmatched | 1.880 | 0.061 | 1.880 | 0.061 | 1.880 | 0.061 |
|  | Matched | -1.380 | 0.169 | -0.990 | 0.324 | -1.340 | 0.183 |
| The proportion of individuals with a middle school or above | Unmatched | 2.740 | 0.006 | 2.740 | 0.006 | 2.740 | 0.006 |
|  | Matched | 0.030 | 0.974 | 0.140 | 0.891 | 0.000 | 0.998 |
| The number of people attended university | Unmatched | 2.640 | 0.009 | 2.640 | 0.009 | 2.640 | 0.009 |
|  | Matched | 0.230 | 0.817 | 0.640 | 0.520 | 0.330 | 0.745 |
| The per capita disposable income | Unmatched | 2.990 | 0.003 | 2.990 | 0.003 | 2.990 | 0.003 |
|  | Matched | -0.100 | 0.921 | -0.110 | 0.914 | -0.180 | 0.857 |
